# Identification of Genes and Long Non-Coding RNAs Putatively Related to *Portunus trituberculatus* Sex Determination and Differentiation Using Oxford Nanopore Technology Full-Length Transcriptome Sequencing

**DOI:** 10.3390/ijms252111845

**Published:** 2024-11-04

**Authors:** Shaoting Jia, Guang Li, Yuchao Huang, Yashi Hou, Baoquan Gao, Jianjian Lv

**Affiliations:** 1 State Key Laboratory of Mariculture Biobreeding and Sustainable Goods, Yellow Sea Fisheries Research Institute, Chinese Academy of Fishery Sciences, Qingdao 266071, China; 2 Laboratory for Marine Fisheries Science and Food Production Processes, Laoshan Laboratory, Qingdao 266237, China; 3 Key Laboratory of Sustainable Development of Marine Fisheries, Ministry of Agriculture and Rural Affairs, Yellow Sea Fisheries Research Institute, Chinese Academy of Fishery Sciences, Qingdao 266071, China

**Keywords:** *Portunus trituberculatus*, ONT sequencing, transcriptome, lncRNA, sex determination and differentiation

## Abstract

The swimming crab (*Portunus trituberculatus*) is an economically important species in China, and its growth traits show obvious sexual dimorphism. Thus, it is important to study the mechanism of sex determination and differentiation in this species. Herein, we identified 2138 differentially expressed genes and 132 differentially expressed long non-coding RNAs (lncRNAs) using Oxford Nanopore Technology full-length transcriptome sequencing. We predicted 561 target genes of the differentially expressed lncRNAs according to their location and base pair complimentary principles. Furthermore, pathways related to sex determination, differentiation, and reproduction were enriched for lncRNAs according to gene ontology and Kyoto Encyclopedia of Genes and Genomes analyses. This indicated that lncRNAs might play regulatory roles in these pathways. Our results could form the basis for future studies of sex determination and differentiation in *P. trituberculatus*.

## 1. Introduction

Sex determination and differentiation are important biological processes in sexually reproductive organisms. In invertebrates, the mechanism of sex determination includes genetic sex determination and environmental sex determination [1]. Genetic sex determination is controlled by genetic factors, such as sex determination genes, whereas environmental sex determination depends on environmental factors, such as temperature, light, and nutritional conditions [2]. It is important to explore and identify sex determination genes to interpret the associated genetic mechanism and for sex-controlled breeding.

Long non-coding RNAs comprise RNAs longer than 200 bp that do not encode a protein [3]. Recently, studies have shown that lncRNAs could regulate sex differentiation by binding different regions of functional genes. In the semi-smooth tongue sole, lncRNA *DMRT2-AS*, which is located near the sex determination gene *dmrt2* (encoding doublesex and Mab-3 related transcription factor 2), could significantly increase the expression level of *dmrt2*, suggesting that lncRNA *DMRT2-AS* is involved in male sex differentiation [4]. In *D. melaonogaster*, lncRNAs *R1S*, *R1A*, and *R2S* could promote the development of primordial germ cells (PGCs) into testes by inhibiting the expression of *sxl* (sex lethal), while the lncRNA *R2A* could promote PGCs to develop into ovaries by activating *sxl* transcription [5]. In silkworms, the lncRNA *BMDSX-AS1* could affect the sex ratio by regulating the alternative splicing of *Bmdsx*, which encodes a critical double sex (dsx) protein that functions downstream of the sex determination cascade [6]. In *Daphnia*, lncRNA *DAPALR* could activate *dsxl* expression (encoding Drosophila sex lethal) by binding to the 5′ untranslated region of the *dsxl* gene to promote male differentiation [7]. The above studies suggested that lncRNAs play important roles in sex determination. Therefore, it would be significant to identify key sex-determination-related lncRNAs using high-throughput sequencing.

The third-generation sequencing technology Oxford Nanopore Technology (ONT) sequencing can generate a read length of greater than 1 Mb based on a single-stranded nucleic acid passing through the *Staphylococcus* α hemolysin 9 (αhl) protein pore [8]. Compared with second-generation transcriptome sequencing, ONT full-length transcriptome sequencing can directly reverse transcribe full-length cDNAs without breaking them into fragments, thus generating ultra-long reads that contain complete transcript sequence information. This method can thus produce more complete and more accurate sequencing information [9,10]. To date, full-length ONT transcriptome sequencing has been successfully used in species including the giant tiger prawn (*Penaeus monodon*) [11], Redfin culter (*Chanodichthys erythropterus*) [12], Chinese tapertail anchovy (*Coilia ectenes*) [13], and Hong Kong grouper (*Epinephelus akaara*) [14].

The swimming crab (*Portunus trituberculatus*) is widely distributed on the southeast coast of China. The growth traits of *P. trituberculatus* show obvious sexual dimorphism, and the growth rate of the male is usually faster than that of the female. Mature female individuals usually have higher market value because of their excellent taste [15]. Therefore, exploring the sex determination mechanism of *P. trituberculatus* is important to establish sex control technology and improve the economic benefits of this species. In our previous study, a high-density genetic linkage map of *P. trituberculatus* was constructed, and the sex determination system was determined as XX/XY according to quantitative trait locus mapping [16]. Furthermore, we identified the sex determination region by a chromosome quotient analysis based on genome and transcriptome data [17]. We also identified *PtDMY* (encoding Doublesex- and mab-3-related transcription factor 1Y) as a candidate gene for *P. trituberculatus* sex determination based on the comparative transcriptomic method and the sex determination region. However, the molecular mechanism of *P. trituberculatus* sex determination remains largely unknown, and no lncRNA related to sex determination has been reported.

Herein, based on our previous study, we conducted ONT full-length transcriptome sequencing of the testes and ovaries of *P. trituberculatus*. We aimed to identify differentially expressed genes (DEGs) and lncRNAs related to sex determination and differentiation. Furthermore, the target genes of the lncRNAs were predicted according to their location and base pair complimentary. The accuracy of the data was verified using quantitative real-time reverse transcription PCR (qRT-PCR). Our results will provide foundation data for the further study of the mechanisms of *P. trituberculatus* sex determination and differentiation.

## 2. Results

### 2.1. De Novo Assembly of the P. trituberculatus Gonad Transcriptome

We obtained 11.56 Gb of raw data from six samples, including three ovary and three testis samples, using ONT single-molecule real-time full-length transcriptome sequencing technology. The raw data were uploaded to the NCBI SRA database (accession No.: SUB14664642). After filtering out low-quality reads, 9,901,034 clean reads were obtained (Table 1). Full-length sequences were identified according to adaptor sequences at both ends of the reads, and the number of full-length sequences obtained per sample varied from 1,074,030 to 1,291,810. All full-length sequences were filtered and aligned to the reference genome, resulting in 40,772 non-redundant transcripts being identified (Table 2).

### 2.2. Identification of DEGs

**Figure 1:**
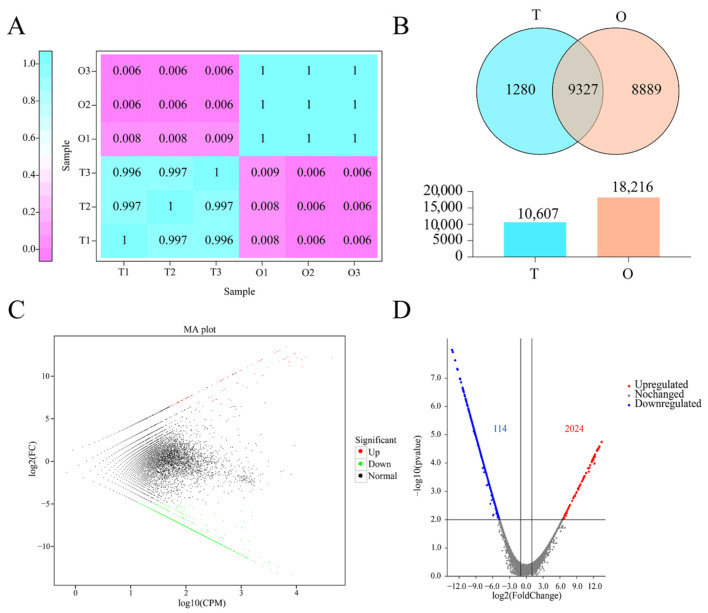
Analysis of the ONT full-length transcriptome sequence of the testis and ovary in *P. trituberculatus*. (**A**) Heatmap of the expression relationship between samples. (**B**) Venn diagram of DEGs between testis and ovary; T: testis, O: ovary. (**C**) MA plots of DEGs. (**D**) Volcano plot of DEGs. ONT, Oxford Nanopore Technology; DEG, differentially expressed gene; MA, M-versus-A; FC, fold change; CPM, counts per million.

The correlations among the three samples from each tissue were high, which indicated that the sequencing results were credible (Figure 1A). We obtained a total of 19,496 genes using ONT full-length transcriptome sequencing between the testis and ovary. There were 1280 genes that were specifically expressed in the testis and 8889 that were specifically expressed in the ovary. In addition, 9327 genes were expressed in both the testis and ovary (Figure 1B). According to the principles of |log2 (fold change)| ≥ 1 and FDR < 0.01, there were a total of 2138 DEGs between the testis and ovary, including 2024 downregulated DEGs and 114 upregulated DEGs (Figure 1C,D). The annotation of the DEGs is shown in Table S4.

### 2.3. Enrichment of GO Terms and KEGG Pathway Analysis of DEGs

**Figure 2:**
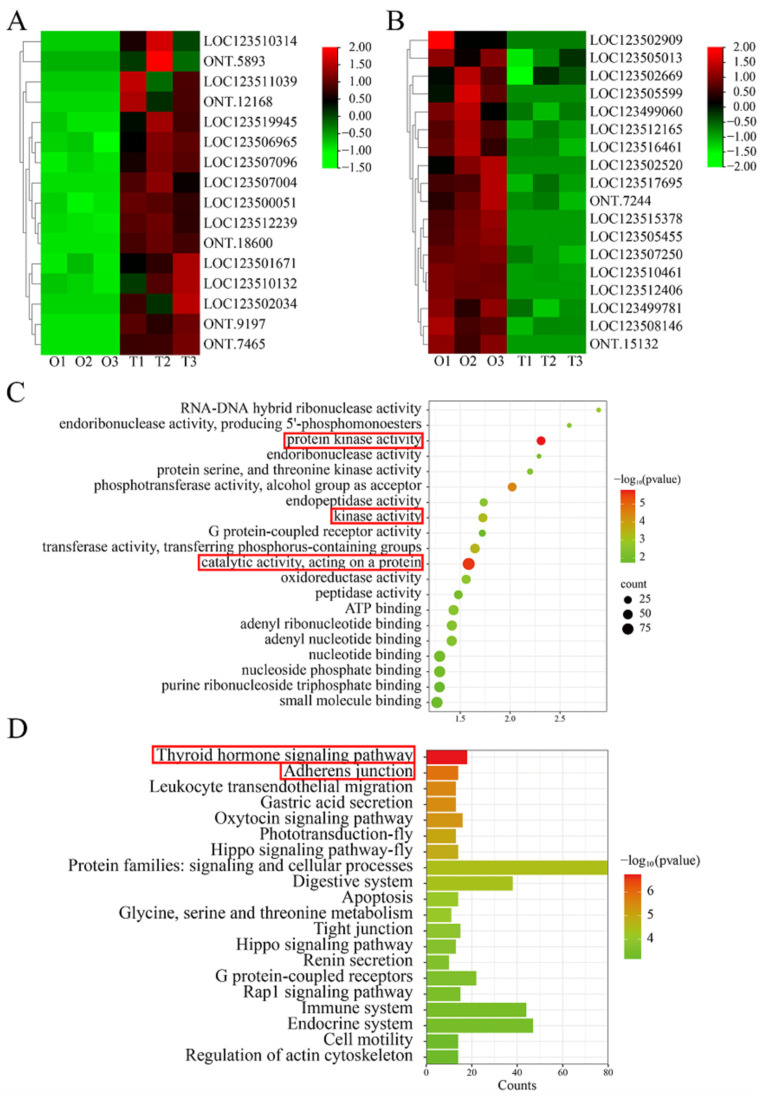
DEGs related to testis and ovary development. (**A**) Heatmap of the top 16 highly expressed DEGs in the testis. (**B**) Heatmap of the top 18 highly expressed DEGs in the ovary. (**C**) Scatter plots of the top 20 enriched biological process gene ontology (GO) terms. (**D**) Column diagram of the top 20 Kyoto Encyclopedia of Genes and Genomes (KEGG) pathways.

The heatmap of the 25 DEGs with the highest expression levels in the testis was shown in Figure 2A, which might be related to spermatogenesis. The 25 DEGs with the highest expression levels in the ovary are shown in Figure 2B, which might be related to oogenesis. The top 20 GO terms are listed in Figure 2C, which are mainly related to cell growth, including protein kinase activity, kinase activity, catalytic activity, and acting on a protein. The top 20 KEGG enrichment pathways for the DEGs are listed in Figure 2D. The DEGs were mainly enriched in the thyroid hormone signaling pathway and adherens junction. The DEGs related to GO terms are shown in Table S5. Furthermore, we performed a polygeny analysis and domain analysis with some key genes related to reproduction, including LOC123500051, LOC123517695, and LOC123510132. The sequences of these genes are relatively conserved and showed high similarity with other species. All of them have conserved functional domains. The results are shown in Figures S1–S3.

### 2.4. Screening for Differentially Expressed lncRNAs

**Figure 3:**
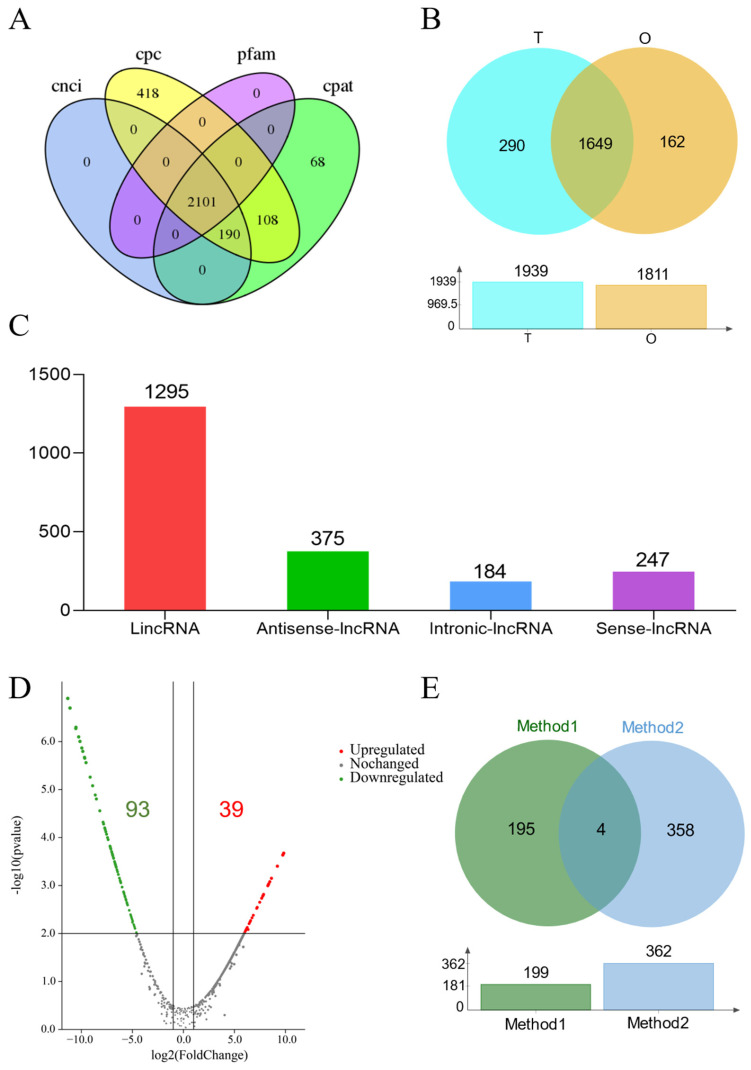
Analysis of lncRNAs from testis and ovary transcriptome sequencing. (**A**) Prediction of lncRNAs by four methods (CPC, CNCI, CPAT, and Pfam). (**B**) Venn diagram of lncRNA expression mode in the testis and ovary; T: testis, O: ovary. (**C**) Four classifications of lncRNAs. (**D**) Volcano plots of differentially expressed lncRNAs created using DESeq2. (**E**) Prediction of lncRNA target genes by two methods. lncRNA, long non-coding RNA; CPC, coding potential calculator; CNCI, coding-non-coding index; CPAT, coding potential assessment tool; Pfam, protein families.

The prediction of lncRNAs using CPC, CNCI, CPAT, and Pfam among the 40,772 transcripts (Figure 3A) obtained 2817, 2201, 2467, and 2101 lncRNAs, respectively. Subsequently, 2101 lncRNAs were confirmed after taking the intersection of the four methods. Among them, 290 lncRNAs were specifically expressed in the testis and 162 were specifically expressed in the ovary (Figure 3B). The other 1649 lncRNAs were expressed both in the testis and ovary. The lncRNAs could be divided into four classifications, long intergenic non-coding RNAs (lincRNAs), antisense lncRNAs, intronic lncRNAs, and sense lncRNAs, according to their location distribution (Figure 3C). DESeq2 identified 162 differentially expressed lncRNAs in the testis compared with the ovary, including 93 downregulated lncRNAs and 39 upregulated lncRNAs (Figure 3D).

### 2.5. Prediction of lncRNA Target Genes and Functional Analysis

**Figure 4:**
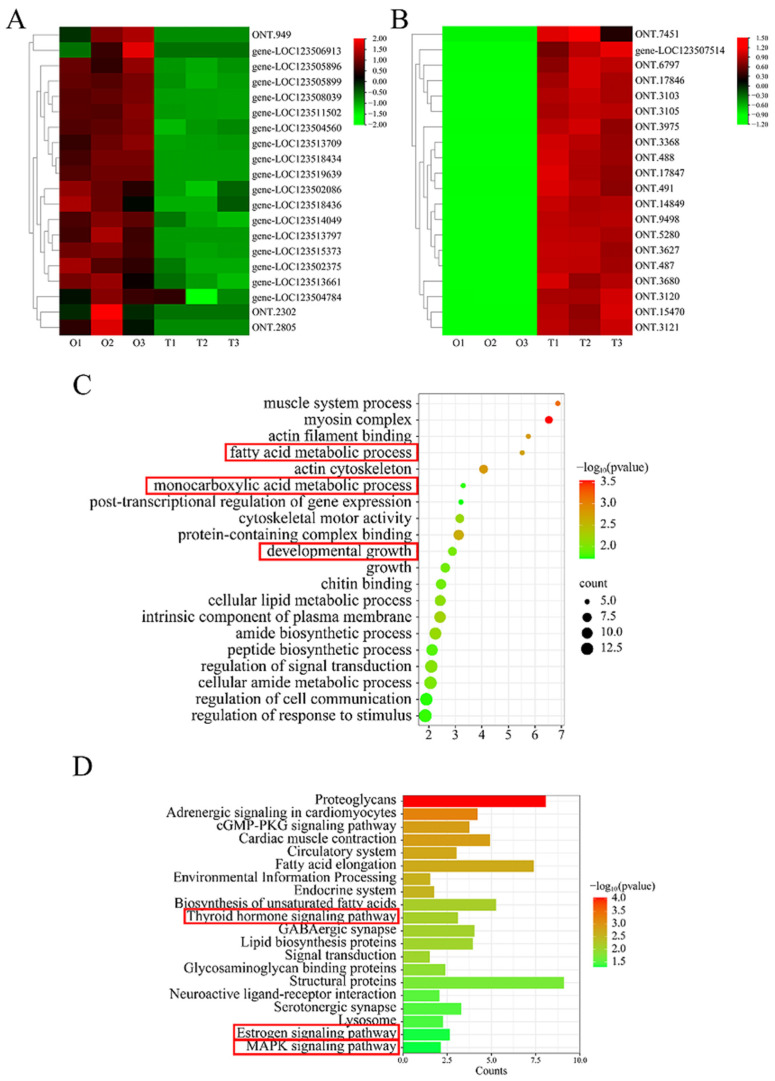
Analysis of the differentially expressed lncRNA target genes. (**A**) Heatmap of the top 20 highly expressed genes in the ovary. (**B**) Heatmap of the top 20 highly expressed genes in the testis. (**C**) Scatter plots of the top 20 enriched biological process GO terms. (**D**) Column diagram of the top 20 KEGG pathways.

**Figure 5:**
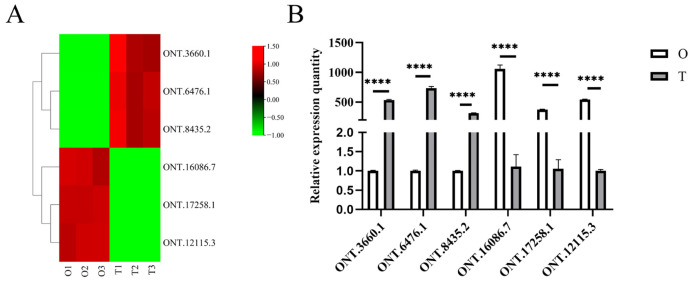
Verification of six differentially expressed lncRNAs. (**A**) Heatmap of six differentially expressed lncRNAs from transcriptome sequencing. (**B**) Validation of the six differentially expressed lncRNAs by qRT-PCR (**** *p* < 0.0001). The data analysis was conducted by the 2^−ΔΔCT^ method, and *β-actin* was chosen as a reference gene.

To further investigate the role of differentially expressed lncRNAs in the gonads, the target genes of the lncRNAs were predicted (Figure 3E) based on the positional relationship between the lncRNA and the gene, mapped in the range of 100 kb upstream and downstream of the lncRNA (Table 3). The other prediction method used base complementary pairing of the gene and lncRNA. By taking the insertion of the two methods, we obtained 557 target genes for the 132 differentially expressed lncRNAs (Table 4). The annotations of the target genes are listed in Tables S2 and S3. The top 20 GO terms of the target genes are shown in Figure 4A. The target genes were mainly enriched in developmental growth, the fatty acid metabolic process, and the monocarboxylic acid metabolic process, which are mainly related to growth and development. The top 20 KEGG pathways related to the target genes are listed in Figure 5, including the estrogen signaling pathway, the thyroid hormone signaling pathway, and the MAPK signaling pathway, which are involved in sex determination and differentiation, as well as gonad development.

### 2.6. Verification by qRT-PCR

To verify the accuracy of the full-length transcriptome data, three testis-specific expressed lncRNAs and three ovary-specific expressed lncRNAs were selected randomly for qRT-PCR validation (Figure 5). The expression levels of these lncRNAs were consistent with the transcriptome data, which indicates the reliability of the sequencing results.

## 3. Discussion

*P. trituberculatus* is an important species with obvious sexual dimorphism in its growth traits. In this study, we conducted ONT full-length transcriptome sequencing on testis and ovary samples to explore genes and lncRNAs related to sex determination and differentiation. We identified 2138 DEGs and 132 differentially expressed lncRNAs. Furthermore, 557 lncRNA target genes were predicted, which were mainly related to sex determination and differentiation, as well as gonad development. The expression level of selected lncRNAs was further verified using qRT-PCR. Our results will help to elucidate the sex determination mechanism in *P. trituberculatus* and provide baseline data for further study.

Currently, several genes related to sex determination and differentiation in *P. trituberculatus* have been reported. For example, the *Ptidmrt1* gene is highly expressed in the testis, and the expression of insulin-like androgenic gland hormone (IAG) showed a significant decrease after RNA-interference-mediated knockdown of *Ptidmrt1*. This result indicated that *Ptidmrt1* might be a potential regulator of IAG [18]. In addition, Jiang et al. found that the expression level of IAG was reduced after knocking down *PtSoxE*, which is a male-specific expressed gene in *P. trituberculatus* encoding SRY-related HMG box E [19]. *lilli-like*, encoding a member of the AF4/FMR2 family, was also found to be related to reproduction by transcriptome sequencing, and is expressed specifically in testis in *P. trituberculatus* [20]. Sex determination and differentiation are important events in animal reproduction, and many genes are involved in these processes [21]. In this study, 2138 DEGs between the testis and ovary were identified comprising 2024 downregulated and 114 upregulated genes, which might be related sex determination and differentiation.

lncRNAs have been reported to play an important regulatory role in sex determination and differentiation in animals. For example, in mammals, lncRNA *XIST* could inactivate the X chromosome by wrapping it, together with other proteins and RNAs [22]. Meanwhile, another antisense lncRNA, *TSIX*, which is located on the X chromosome, might also be involved in X chromosome silencing [23]. In birds, a 9 kb lncRNA transcribed from a male hypermethylated region of the Z chromosome could affect the expression of nearby genes in males and females, with a certain metrological compensatory effect [24,25]. In the rice eel, an lncRNA could form a network with mRNA to regulate sex differentiation by promoting the expression of *cyp19a1a* (encoding cytochrome P450 family 19 subfamily A member 1) [26]. In *P. trituberculatus*, there have been no reports related to the roles of lncRNAs in sex determination and differentiation. Herein, we identified 132 lncRNAs that were expressed differentially between the testis and ovary (93 downregulated and 39 upregulated). The target genes of these lncRNAs were also predicted (Table 3 and Table 4). We speculated that these lncRNAs might be related to sex determination and differentiation in *P. trituberculatus*.

In conclusion, DEGs and differentially expressed lncRNAs between the testis and ovary in *P. trituberculatus* were identified among data generated using ONT full-length transcriptome sequencing. GO and KEGG analysis of the differentially expressed genes and lncRNAs identified the enrichment of pathways related to sex determination and differentiation. Consequently, this study laid the foundation for subsequent studies of genes and lncRNAs involved in sex determination and differentiation in *P. trituberculatus*.

## 4. Materials and Methods

### 4.1. Ethics

This study was reviewed and approved by the Ethics Committee of the Yellow Sea Fisheries Research Institute. All experiments were performed in accordance with the Guidelines for the Care and Use of Laboratory Animals in China. This study was approved by the Institutional Animal Care and Use Committee of the Yellow Sea Fisheries Research Institute, Chinese Academy of Fishery Sciences (Qingdao, China; approval No.: ACUC-20190314; 15 June 2023).

### 4.2. Animals and Sample Preparation

The *P. trituberculatus* used in this study were cultured by WeiFang Changyi Aquaculture Co., Ltd. (Weifang, China). The ovaries and testes were taken from live and healthy adult *P. trituberculatus*, with three biological replicates of each tissue. Total RNA was extracted using TRIzol (Invitrogen, Carlsbad, CA, USA) according to the manufacturer’s instructions. The RNA integrity was assessed using 1.0% agarose gel electrophoresis, and the RNA concentration was determined using a UV spectrophotometer (Nanodrop 2000 Thermo Fisher Scientific, Waltham, MA, USA). Each RNA sample was used to create an independent sequencing library.

### 4.3. cDNA Library Construction and Sequencing

The full-length cDNA libraries were constructed using a cDNA-PCR sequencing kit (SQK-LSK110 + EXP-PCB096) according to manufacturer’s instructions. The sequencing adaptor was added to both ends of the first-strand cDNA. Double-stranded cDNA was then synthesized employing LongAmp Tag (Ipswich, MA, USA) using 14 cycles of PCR, and the product was purified using Agencourt XP beads (Beckman, Indianapolis, IN, USA). The final cDNA library was added to the flowcells (FLO-PRO002), followed by sequencing on the PromethION platform at Biomarker Technology Company (Beijing, China).

### 4.4. De Novo Assembly and Annotation

The raw data in fast5 format were converted to fastq format using Guppy software (MinKNOW2.2). The total clean data were obtained after filtering out short fragments and low-quality sequences using NanoFilt software (v2.8.0). Then, ribosomal RNA sequences were filtered out after being aligned with public databases. Subsequently, the full-length sequences were identified according to the sequencing adaptors at both ends of the reads. The full-length sequences were analyzed using Stringtie software (v2.2.3) to obtain the consensus sequences. The consensus sequences were then aligned to the reference genome using Minimap2 software (v2.16) [17]. The transcripts were functionally annotated using six databases: Non-redundant (NR), protein families (Pfam), eukaryotic orthologous groups (KOG)/Clusters of Orthologous Groups (COG), evolutionary genealogy of genes: Non-supervised Orthologous Groups (eggNOG), Swiss-Prot, the Kyoto Encyclopedia of Genes and Genomes (KEGG), and gene ontology (GO).

### 4.5. DEG Analysis and Prediction of lncRNA Target Genes

The lncRNA transcripts were predicted using four methods, the coding potential calculator (CPC, 0.9-r2), coding-non-coding index (CNCI, v2), coding potential assessment tool (CPAT, v1.2.2), and Pfam protein domain analysis, and the combined results of the four methods determined whether a sequence was an lncRNA or not. The expression levels of transcripts were calculated using the CPM (counts per million) method [27], and the differential expression analysis between groups was performed using DESeq2 (v1.6.3), with |log2 (fold change)| ≥ 1 and a false discovery rate (FDR) < 0.01 as the screening criteria.

Two methods were used to predict the target genes of the differentially expressed lncRNAs. The first was based on the distance between the lncRNA and the target gene on the chromosome, which was limited to the range of 100 kb upstream and downstream of the lncRNA. The second method used the LncTar software (v1.0) according to the base complementary pairing of the target gene and the lncRNA [28]. Genes predicted by both methods were identified as the target genes of the lncRNAs. The identified target genes were then subjected to GO and KEGG enrichment analysis.

### 4.6. qRT-PCR

Total RNA was extracted from testes and ovaries using TRIzol according to the manufacturer’s instructions. After the genomic DNA was removed using DNaseI, the cDNA was synthesized using a Primescript™ RT reagent kit (Takara Bio, Dalian, China). The quantitative real-time PCR step to analyze the gene expression level was carried out using the cDNA as the template with TB green premix ex (Takara Bio) according to the manufacturer’s instructions. The reaction comprised 2 × Terra PCR direct TB green premix, 10 μL; 0.4 μL of upstream and downstream primers; cDNA, 2 μL; Rox reference dye lsr, 2 μL; and sterile PCR-grade water, 5.2 μL. The amplification conditions were as follows: 95 °C for 3 min, followed by 40 cycles at 95 °C for 5 s and 60 °C for 34 s. The primers used are listed in Table S1. The data analysis was conducted using the 2^−ΔΔCT^ method, and the gene encoding β-actin was used as the reference gene [29].

## Figures and Tables

**Table 1:** Statistics of the clean data.

Sample ID	ReadNum	BaseNum	N50	MeanLength	MaxLengtth	MeanQecore
O1	1,681,233	2,326,628,468	1544	1383	52,833	Q12
O2	1,739,527	2,380,497,372	1526	1368	257,809	Q12
O3	1,705,668	2,343,230,079	1535	1373	35,629	Q12
T1	1,741,545	2,148,691,004	1446	1233	133,435	Q12
T2	1,492,049	1,905,251,378	1514	1276	732,368	Q12
T3	1,541,012	1,987,709,609	1531	1289	259,653	Q12

O1, ovary sample 1; O2, ovary sample 2; O3, ovary sample 3; T1, testis sample 1; T2, testis sample 2; T3, testis sample 3; ReadNum, number of reads; BaseNum, number of bases; N50, the sequence length of the shortest contig at 50% of the total assembly length.

**Table 2:** Statistics of the full-length reads.

Sample ID	Number of Clean Reads (Except rRNA)	Number of Full-Length Reads	Full-Length (FL) Percentage
O1	1,478,163	1,255,343	84.93%
O2	1,527,329	1,291,810	84.58%
O3	1,514,212	1,285,096	84.87%
T1	1,504,139	1,281,012	85.17%
T2	1,276,544	1,074,030	84.14%
T3	1,312,047	1,102,977	84.07%

**Table 3:** Prediction of lncRNA target genes according to positional relationships.

lncRNA ID	Target mRNA ID
ONT.2462.6	gene-LOC123502386; gene-LOC123502391; gene-LOC123502393; ONT.2463
ONT.11194.6	gene-LOC123512685; gene-LOC123512668; gene-LOC123512745; gene-LOC123512747; gene-LOC123512748; ONT.11342
ONT.19505.7	gene-LOC123498782; gene-LOC123498569; gene-LOC123498497; gene-LOC123498539; gene-LOC123498462; gene-LOC123498731; gene-LOC123498498; gene-LOC123498715; ONT.19137; ONT.19507; ONT.19500; ONT.19503; ONT.19138
ONT.14174.1	gene-LOC123516278; gene-LOC123516276; gene-LOC123515882; gene-LOC123516274; ONT.14173;
ONT.17863.2	ONT.17864
ONT.3032.5	gene-LOC123502950; gene-LOC123502953; gene-LOC123502951; ONT.2876
ONT.10087.1	gene-LOC123511547; gene-LOC123511639; gene-LOC123511626; gene-LOC123511533; gene-LOC123511579; gene-LOC123511582; gene-LOC123511578; ONT.10088
ONT.17683.1	gene-LOC123520288; gene-LOC123520285; gene-LOC123520286
ONT.17381.1	gene-LOC123519227; gene-LOC123519563; gene-LOC123519228; gene-LOC123519564; gene-LOC123519557; gene-LOC123519561; gene-LOC123519566; gene-LOC123519560; gene-LOC123519558; gene-LOC123519229; gene-LOC123519556; ONT.17199; ONT.17383; ONT.17384; ONT.17380; ONT.17382; ONT.17200

**Table 4:** Prediction of lncRNA target genes according to base complementary.

lncRNA ID	Target mRNA ID
ONT.6543.1	gene-LOC123507132
ONT.2969.4	gene-LOC123513843; ONT.1615
ONT.10381.4	gene-LOC123511406
ONT.16305.4	ONT.3690; ONT.689
ONT.17224.7	ONT.8463; ONT.16135; ONT.1598; ONT.3690; ONT.3820; ONT.689
ONT.18694.2	ONT.12196; ONT.6220; ONT.794; ONT.18980; ONT.15683; ONT.19157; ONT.10382; ONT.4590; ONT.16436
ONT.3372.1	ONT.14093; ONT.15765; ONT.14384; ONT.15470; ONT.14415
ONT.10956.1	gene-LOC123512250
ONT.17281.2	gene-LOC123519669

## Data Availability

Data is contained within the article and Supplementary Materials.

## Supplementary Materials

The following supporting information can be downloaded at https://www.mdpi.com/article/10.3390/ijms252111845/s1.
