# Supplementary material for: Identification of Genes and Long Non-Coding RNAs Putatively Related to Portunus trituberculatus Sex Determination and Differentiation Using Oxford Nanopore Technology Full-Length Transcriptome Sequencing
Source: Int J Mol Sci. 2024 Nov 4;25(21):11845. doi: 10.3390/ijms252111845 (PMC11546564; doi:10.3390/ijms252111845)
Supplement: Supplementary file 1 [file ijms-25-11845-s001.zip › Supplementary material-Figure S1-S3.pdf]

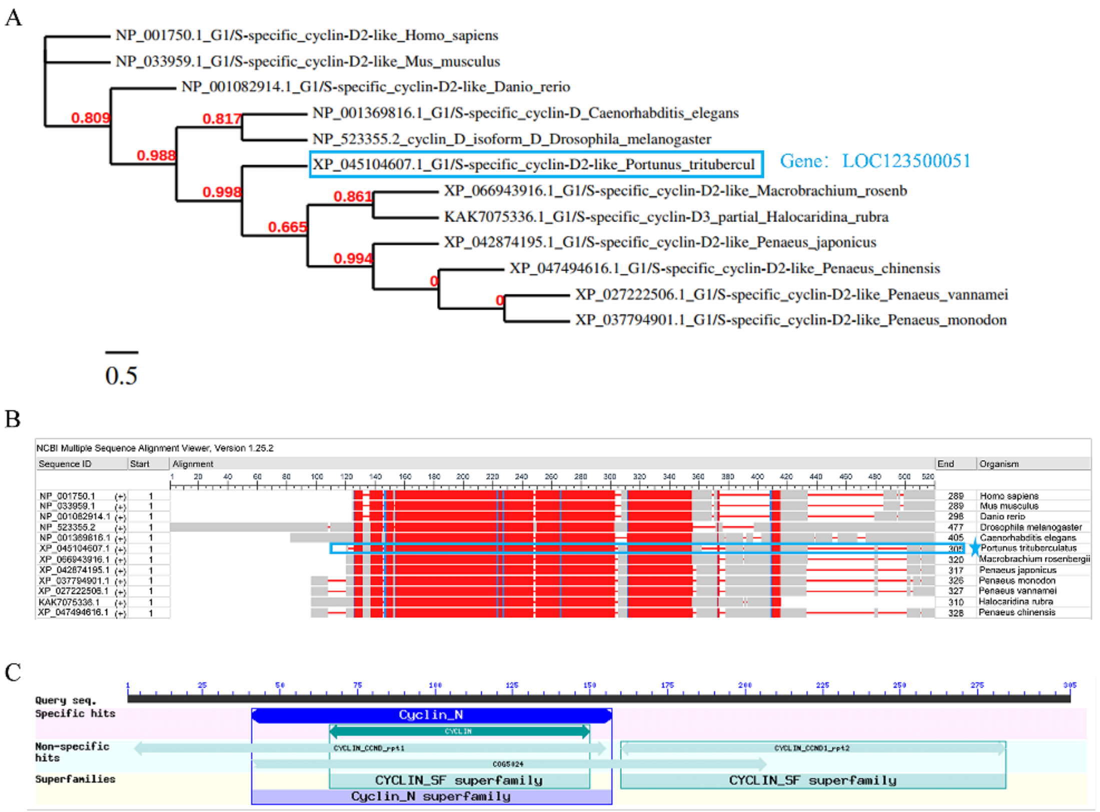

Figure S1. The phylogeny analysis and conserved domain analysis of LOC123500051. A. The evolutionary tree analysis of LOC123500051. B. The multiple sequence alignments of LOC123500051 with other species. C. The conserved domain analysis of LOC123500051.

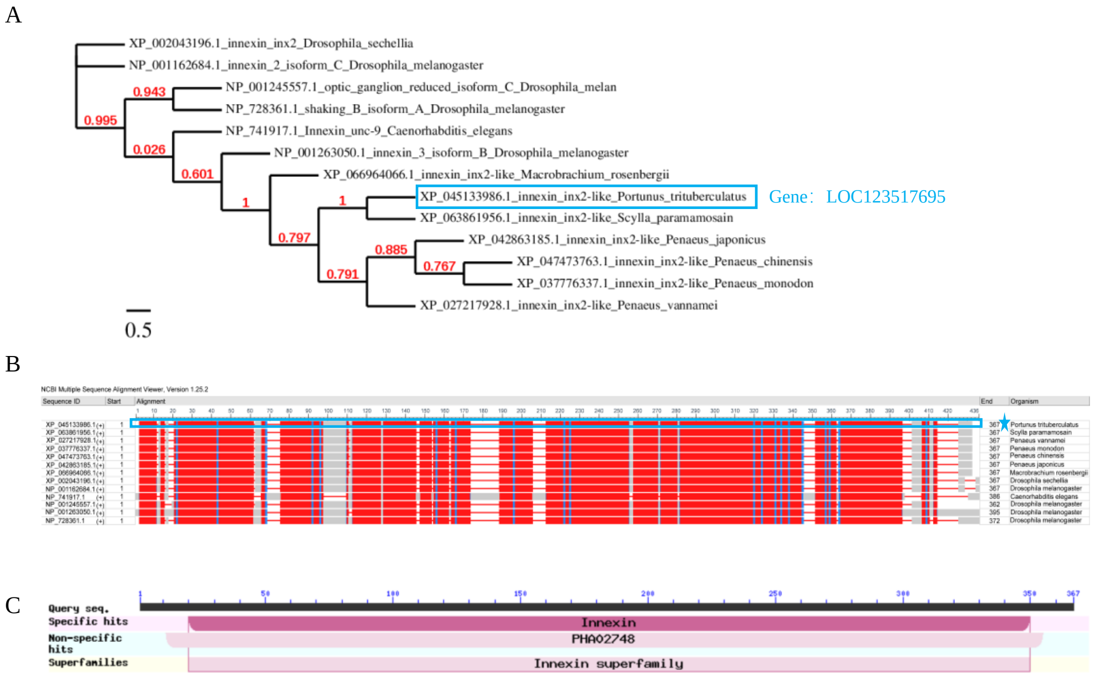

Figure S2. The phylogeny analysis and conserved domain analysis of LOC123517695. A. The evolutionary tree analysis of LOC123517695. B. The multiple sequence alignments of LOC123517695 with other species. C. The conserved domain analysis of LOC123517695.

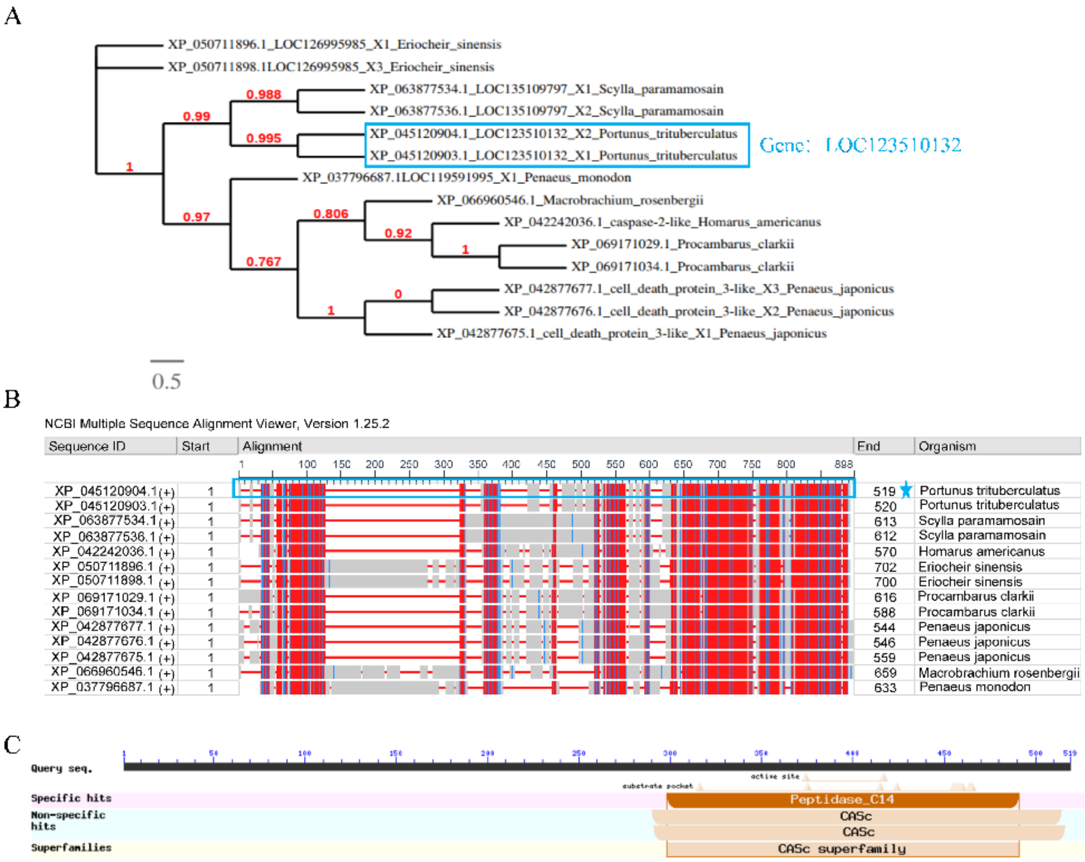

Figure S3. The phylogeny analysis and conserved domain analysis of LOC123510132. A. The evolutionary tree analysis of LOC123510132. B. The multiple sequence alignments of LOC123510132 with other species. C. The conserved domain analysis of LOC123510132.
