# Supplementary material for: Identification of Genes and Long Non-Coding RNAs Putatively Related to Portunus trituberculatus Sex Determination and Differentiation Using Oxford Nanopore Technology Full-Length Transcriptome Sequencing
Source: Int J Mol Sci. 2024 Nov 4;25(21):11845. doi: 10.3390/ijms252111845 (PMC11546564; doi:10.3390/ijms252111845)
Supplement: Supplementary file 1 [file ijms-25-11845-s001.zip › Supplementary material-Tables S1-S3.pdf]

Table S1. Prime sequence

Primer Name	Primer Sequence (5’-3’)	Purpose
ONT.12115.3-F	CTGCCACTACTGGAGAGAGAAC	*real-time PCR*
ONT.12115.3-R	GGACAGTATGCGTCTTCGTT	
ONT.17258.1-F	GCTATGTAGTCGTGGTGGGTGA	
ONT.17258.1-R	CGATTGCTTCCCCACTATTCTC	
ONT.3660.1-F	CCCCTCTCGGAACTCACAAA	
ONT.3660.1-R	CTTACATGCCTGCCTTGCTC	
ONT.6476.1-F	TTGGAGTTTGGAGTGAGGGA	
ONT.6476.1-R	AGAGGATGCGCGTTGAAGTT	
ONT.8435.2-F	CTCCTTTACCTCCTCATTCAGA	
ONT.8435.2-R	GGCAGTATCTGGTGGTTGGT	
ONT.16086.7-F	TGCCACTACTGAAGGAGAATCG	
ONT.16086.7-R	CCTCCGAGGAAGTGTAGCTTTG	
β-actin-F	CGAAACCTTCAACACTCCCG	
β-actin-R	GGGACAGTGTGTGAAACGCC	

Table S2. Description of predicted target genes according to position

**LncRNA ID**	**Target mRNA ID**	**Description**
ONT.2462.6	gene-LOC123502386	uncharacterized
	gene-LOC123502391	vegetative cell wall protein gp1-like
	gene-LOC123502393	uncharacterized
	ONT.2463	new discovered
ONT.11194.6	gene-LOC123512685	serine/threonine-protein kinase OSR1-like
	gene-LOC123512668	glutathione S-transferase theta-1-like
	gene-LOC123512745	diaphanous related formin 1
	gene-LOC123512747	sec1 family domain-containing protein 2-like

	gene-LOC123512748	allantoicase-like
	ONT.11342	new discovered
ONT.19505.7	gene-LOC123498782	uncharacterized
	gene-LOC123498569	protein TsetseEP-like
	gene-LOC123498497	roundabout homolog 3-like
	gene-LOC123498539	uncharacterized
	gene-LOC123498462	uncharacterized
	gene-LOC123498731	uncharacterized
	gene-LOC123498498	histone-lysine N-methyltransferase, H3 lysine-79 specific-like
	gene-LOC123498715	uncharacterized
	ONT.19137	new discovered
	ONT.19507	new discovered
	ONT.19500	new discovered
	ONT.19503	new discovered
	ONT.19138	new discovered
ONT.14174.1	gene-LOC123516278	splicing factor YJU2-like
	gene-LOC123516276	uncharacterized
	gene-LOC123515882	uncharacterized
	gene-LOC123516274	reactive oxygen species modulator 1-like
	ONT.14173	new discovered
ONT.17863.2	ONT.17864	new discovered
ONT.3032.5	gene-LOC123502950	glycoprotein gp100-like
	gene-LOC123502953	cysteine-rich hydrophobic domain-containing protein 2-like
	gene-LOC123502951	cysteine-rich hydrophobic domain-containing protein 2-like
	ONT.2876	new discovered
ONT.10087.1	gene-LOC123511547	glycerophosphocholine cholinephosphodiesterase ENPP6-like
	gene-LOC123511639	troponin C, isotype gamma-like
	gene-LOC123511626	eisosome protein SEG2-like
	gene-LOC123511533	alpha-tocopherol transfer protein-like
	gene-LOC123511579	myosin type-2 heavy chain 2-like
	gene-LOC123511582	uncharacterized
	gene-LOC123511578	uncharacterized
	ONT.10088	new discovered
ONT.17683.1	gene-LOC123520288	uncharacterized
	gene-LOC123520285	60S ribosomal protein L22-like
	gene-LOC123520286	vesicle-associated membrane protein 2-like
ONT.17381.1	gene-LOC123519227	mucin-2-like
	gene-LOC123519563	glycerophosphocholine cholinephosphodiesterase ENPP6-like

	gene-LOC123519228	mucin-2-like
	gene-LOC123519564	pre-mRNA-splicing factor ISY1 homolog
	gene-LOC123519557	coiled-coil domain-containing protein 115-like
	gene-LOC123519561	CUE domain-containing protein 2-A-like
	gene-LOC123519566	cytochrome c oxidase assembly factor 5-like
	gene-LOC123519560	mucin-17-like
	gene-LOC123519558	Transmembrane protein Pmi
	gene-LOC123519229	NADH dehydrogenase (ubiquinone) complex I, assembly factor 6 homolog sicily
	gene-LOC123519556	Heat shock protein 70 cognate 5
	ONT.17199	new discovered
	ONT.17383	new discovered
	ONT.17384	new discovered
	ONT.17380	new discovered
	ONT.17382	new discovered
	ONT.17200	new discovered

Table S3. Description of predicted target genes according to base complementary

**LncRNA ID**	**Target mRNA ID**	**Description**
ONT.6543.1	gene-LOC123507132	formin-like protein 14
ONT.2969.4	gene-LOC123513843	deoxyribose-phosphate aldolase-like
	ONT.1615	new discovered
ONT.10381.4	gene-LOC123511406	uncharacterized
ONT.16305.4	ONT.3690	new discovered
	ONT.689	new discovered
ONT.17224.7	ONT.8463	new discovered
	ONT.16135	new discovered
	ONT.1598	new discovered
	ONT.3690	new discovered
	ONT.3820	new discovered
	ONT.689	new discovered

ONT.18694.2	ONT.12196	new discovered
	ONT.6220	new discovered
	ONT.794	new discovered
	ONT.18980	new discovered
	ONT.15683	new discovered
	ONT.19157	new discovered
	ONT.10382	new discovered
	ONT.4590	new discovered
	ONT.16436	new discovered
ONT.3372.1	ONT.14093	new discovered
	ONT.15765	new discovered
	ONT.14384	new discovered
	ONT.15470	new discovered
	ONT.14415	new discovered
ONT.10956.1	gene-LOC123512250	KH domain-containing protein akap-1-like
ONT.17281.2	gene-LOC123519669	eIF-2-alpha kinase activator GCN1-like
